# A case of renal cell carcinoma with antiphospholipid syndrome treated by robot‐assisted partial nephrectomy

**DOI:** 10.1002/iju5.12654

**Published:** 2023-10-18

**Authors:** Yu Tashiro, Satoshi Ishitoya, Ryo Yamamoto, Kyohei Sugiyama, Hideaki Takada, Keiyu Matsumoto, Kazunari Tsuchihashi

**Affiliations:** ^1^ Department of Urology Otsu Red Cross Hospital Otsu Shiga Japan; ^2^ Department of Urology Kyoto University Graduate School of Medicine Sakyo‐Ku Kyoto Japan

**Keywords:** antiphospholipid syndrome, antithrombotic drug, nephrectomy, renal cell carcinoma, robot‐assisted surgery

## Abstract

**Introduction:**

Antiphospholipid syndrome is an autoimmune disease that presents with thrombus hyperplasia. Although very rare, this disease is reported to become severe after the surgical invasion and other interventions. To our knowledge, there are no reports of partial nephrectomy in patients with antiphospholipid.

**Case presentation:**

A 45‐year‐old man visited our hospital for treatment of left renal cell carcinoma. He had a history of antiphospholipid syndrome and took two antithrombotic agents. We performed a robot‐assisted partial nephrectomy. We selectively ligated only the feeding branch during the procedure. Postoperatively, there were no complications, and the patient was discharged on postoperative day 10. One year after surgery, there was no worsening of antiphospholipid syndrome.

**Conclusion:**

We reported the first case of robot‐assisted partial nephrectomy for an antiphospholipid syndrome patient. Selective ligation of the renal artery might not have contributed to the severe antiphospholipid syndrome.

**Abbreviations & Acronyms**
APS: antiphospholipid antibody syndrome
CAPS: catastrophic antiphospholipid syndrome
CT: computed tomography
RCC: renal cell carcinoma
RAPN: robot‐assisted partial nephrectomy

**Keynote message**

We report the first case of renal cell carcinoma patient with antiphospholipid antibody syndrome treated by robot‐assisted partial nephrectomy. Our intraoperative devise might result in an uncomplicated outcome.

## Introduction

APS is an autoimmune disease with antibodies against phospholipid‐binding proteins that present with clinical symptoms such as venous or arterial thrombosis, recurrent miscarriage, and thrombocytopenia.^1^ To our knowledge, there are no reports of partial nephrectomy for RCC complicated by APS. In this study, we report a case of RCC with APS treated by RAPN, with a literature review.

## Case presentation

**Fig. 1:**
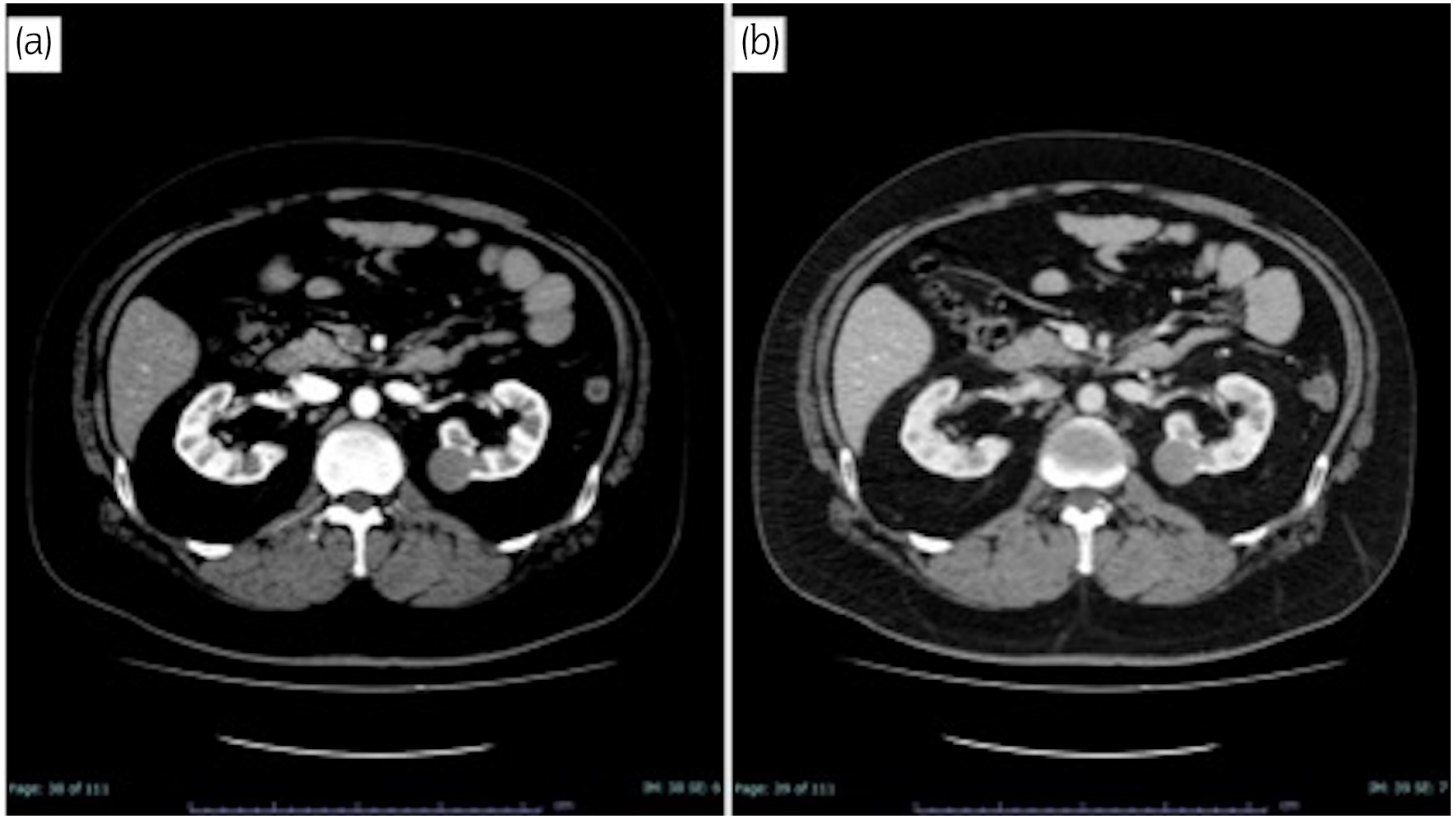
Abdominal dynamic CT. A well‐defined mass with a diameter of 27 mm was seen on the medial dorsal side of the left kidney with a weak contrast effect in the early phase (a) and a progressive increase in contrast effect in the equilibrium phase (b).

**Fig. 2:**
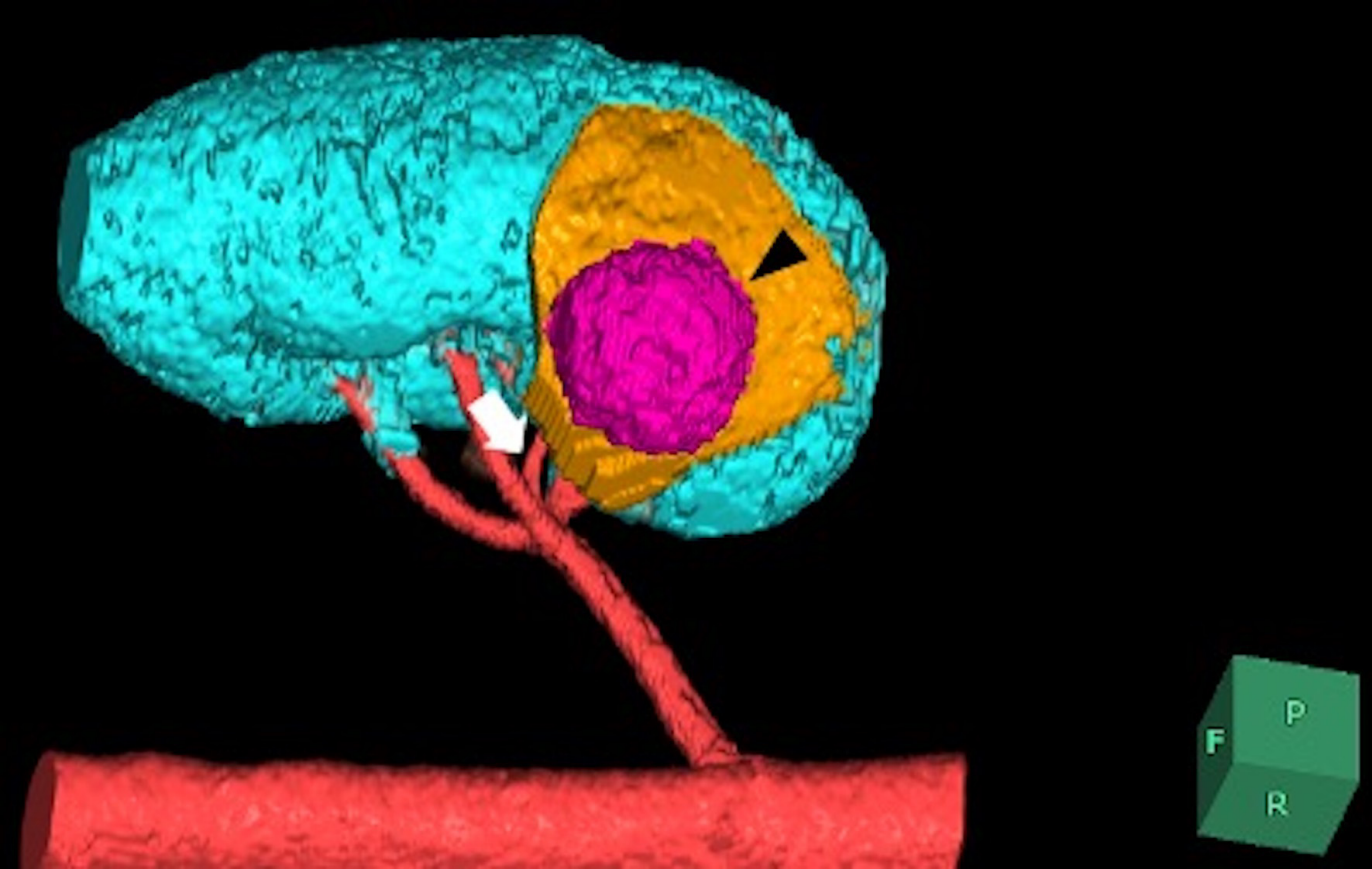
3D reconstruction of the CT scan. The renal tumor (black arrow) is seen on the dorsal of the kidney. The feeding branch (white arrow) entered the tumor directly from the first posterior branch of the renal artery and the ischemic area (yellow area) was identified.

A 45‐year‐old man visited our hospital for an examination of a left kidney tumor, which was found asymptomatically during an examination for upper respiratory tract infection. He had a history of APS, chronic kidney disease (creatinine 1.79 mg/dL), deep vein thrombosis, and epileptic seizures and was taking aspirin, edoxaban, lamotrigine, and clobazam. Contrast‐enhanced CT showed a well‐defined, contrast‐enhanced mass of 23 mm in the central dorsal aspect of the left kidney. Because renal angiomyolipoma could not be ruled out, CT‐guided biopsy was performed, but no definitive diagnosis was made. Three months after the initial CT, the mass had increased to 27 mm on CT (Fig. 1a,b); therefore, we diagnosed nonclear RCC cT1aN0M0 (RENAL nephrometry score: 1 + 1 + 1 + p + 2 = 5p). We planned RAPN because we considered that the advantages of partial nephrectomy outweighed those of radical nephrectomy, even under APS complications, based on age and renal function, in addition to the technical feasibility of the procedure. Two antithrombotic drugs were replaced with heparin. A minor decrease in platelet count to 51 000/μL was observed after the start of heparin administration. The patient had only minor gingival bleeding and no other bleeding tendency. Heparin administration was stopped 6 h before surgery. We started RAPN via the retroperitoneal approach using the da Vinci Xi surgical system® (Intuitive Surgical, Sunnyvale, CA, USA). We could selectively ligate the feeding artery (Fig. 2) without clamping the main renal artery trunk 6 (Only taping was performed in case of bleeding) because we were able to identify the posterior branch supplying the tumor, which had been identified on preoperative imaging using the SYNAPSE VINCENT® (Fujifilm Medical Co. Ltd., Tokyo, Japan) (Fig. 2). Since there was little parenchymal defect, urinary release and arterial bleeding were not observed, we did not perform inner suture or outer suture, but only hemostasis of the resected surface of the renal parenchyma. The operating time was 251 min, the console time was 105 min, no warm ischemic time, and the blood loss was 150 mL. During the procedure, we transfused 10 units of concentrated platelet solution to prevent posterior bleeding. After surgery, the patient was admitted to the intensive care unit, and heparin administration was resumed. No bleeding tendency was observed after the restart of heparin administration. The patient restarted antithrombotic agents on postoperative day 1. Postoperatively, there were no signs of a tendency for bleeding or thrombus formation, and blood tests on postoperative day 7 showed a platelet count of 75 000/μL and creatinine of 1.86 mg/dL. Contrast‐enhanced CT on postoperative day 8 showed no new thrombus or hematoma, and the patient was discharged on postoperative day 10. The pathological result of the renal tumor was mucinous tubular and spindle cell carcinoma. One year after surgery, the patient's creatinine level was 1.6 mg/mL, and there was no recurrence of RCC or worsening of APS.

## Discussion

APS is an autoimmune disease characterized by frequent and recurrent arteriovenous thromboses and habitual abortions triggered by the presence of antiphospholipid antibodies.^1^ APS sometimes leads to catastrophic status (CAPS) characterized by multiple organ failure. Although the pathogenesis of CAPS is still unclear, a two‐hit hypothesis has been assumed. That is, patients who are likely to form a thrombus are inclined to activate their coagulation system and form a thrombus due to the withdrawal of antithrombotic drugs, surgical invasion, infection, or other triggers. The thrombus may cause organ ischemia, which in turn leads to further thrombosis.^2^ Although heparin, corticosteroids, and plasma exchange have been recommended as treatment options, the treatment procedure for CAPS has not yet been established.^3^

Therefore, we considered that it was important to avoid developing CAPS and that partial nephrectomy, which involves clamping and unclamping the blood vessels, may have been a risk for developing CAPS. In this case, the 3D reconstruction image showed in detail that one posterior branch of the renal artery was supplying the tumor (Fig. 2).To prevent intra‐arterial thrombosis and reduce the risk of CAPS, we planned to ligate the feeding branch of the renal artery selectively without interrupting the main renal artery. Intraoperatively, we identified the feeding artery and completed the procedure without clamping the main renal artery. The perioperative platelet count and D‐dimer level were stable, and there were no signs of new thrombus formation.

From the view of renal artery ischemia and reperfusion, we reviewed reports of living donor kidney transplantation in patients with end‐stage renal failure complicated by APS. Although thrombotic microangiopathy occurred in the transplanted kidney and graft function was delayed, there was one report that APS did not become severe.^4^ However, corticosteroids and plasma exchange were administered, and the possibility that organ ischemia–reperfusion could contribute to the worsening of APS could not be ruled out.

To the best of our knowledge, there have been some reports of nephrectomy for RCC complicated with APS, but this is the first report of partial nephrectomy for RCC complicated with APS.^5^ Although the safety of total clamping of the renal artery is still unclear, we could perform the operation safely by selectively ligating the feeding branch.

## Author contributions

Yu Tashiro: Writing – original draft. Satoshi Ishitoya: Supervision. Ryo Yamamoto: Resources. Kyohei Sugiyama: Resources. Hideaki Takada: Resources. Keiyu Matsumoto: Resources. Kazunari Tsuchihashi: Resources.

## Conflict of interest

The authors declare no conflict of interest.

## Approval of the research protocol by an Institutional Reviewer Board

Not applicable.

## Informed consent

Informed consent was obtained from the patient for the publication of this case report.

## Registry and the Registration No. of the study/trial

Not applicable.
